# Automated Cerebral Infarct Detection on Computed Tomography Images Based on Deep Learning

**DOI:** 10.3390/biomedicines10010122

**Published:** 2022-01-06

**Authors:** Syu-Jyun Peng, Yu-Wei Chen, Jing-Yu Yang, Kuo-Wei Wang, Jang-Zern Tsai

**Affiliations:** 1 Professional Master Program in Artificial Intelligence in Medicine, College of Medicine, Taipei Medical University, 19F, No. 172-1, Sec. 2, Keelung Rd., Taipei City 10675, Taiwan; sjpeng2019@tmu.edu.tw; 2 Department of Neurology, Landseed International Hospital, No. 77, Guang-Tai Rd., Taoyuan 324609, Taiwan; 3 Department of Neurology, National Taiwan University Hospital, No. 7, Zhong-Shang South Rd., Taipei 10002, Taiwan; 4 Department of Electrical Engineering, National Central University, No. 300, Jung-Da Rd., Taoyuan 320317, Taiwan; adam106521131@g.ncu.edu.tw; 5 Department of General Affairs, Landseed International Hospital, No. 77, Guang-Tai Rd., Taoyuan 324609, Taiwan; wangkw@landseed.com.tw

**Keywords:** computed tomography, cerebral infarct detection, acute ischemic stroke, artificial intelligence, deep learning

## Abstract

The limited accuracy of cerebral infarct detection on CT images caused by the low contrast of CT hinders the desirable application of CT as a first-line diagnostic modality for screening of cerebral infarct. This research was aimed at utilizing convolutional neural network to enhance the accuracy of automated cerebral infarct detection on CT images. The CT images underwent a series of preprocessing steps mainly to enhance the contrast inside the parenchyma, adjust the orientation, spatially normalize the images to the CT template, and create a t-score map for each patient. The input format of the convolutional neural network was the t-score matrix of a 16 × 16-pixel patch. Non-infarcted and infarcted patches were selected from the t-score maps, on which data augmentation was conducted to generate more patches for training and testing the proposed convolutional neural network. The convolutional neural network attained a 93.9% patch-wise detection accuracy in the test set. The proposed method offers prompt and accurate cerebral infarct detection on CT images. It renders a frontline detection modality of ischemic stroke on an emergent or regular basis.

## 1. Introduction

Shortening the time from onset to treatment is critical for improving the prognosis of acute ischemic strokes. In the era of reperfusion therapy for acute ischemic strokes, the capability of quick detection of cerebral infarct and a prompt referral from a busy emergency room to a neurology specialist for thrombolysis or thrombectomy is important [1].

The computed tomography (CT) has been the first-line diagnostic modality for patients who are suspected to have an acute stroke [2]. Among all strokes, approximately 87% are ischemic and the rest are hemorrhagic [3]. On CT images, the presence of intracranial hemorrhage can be easily detected. However, detecting cerebral infarct due to ischemic stroke has not been easy. It is because lesion boundaries of ischemic strokes in CT images are not clearly defined [4]. Both CT and magnetic resonance imaging (MRI) can be used for delineation of brain stroke lesions [5,6]. In the recent two decades, researchers have been devising automated cerebral infarct delineation methods to allow for operator-independence, reproducibility, and a considerable saving of detection time [5,7,8]. Due to the relatively lower image contrast [9], accurate delineation with CT poses more challenges than with MRI.

The traditional algorithms for automated infarct delineation were devised on the basis of a set of feature extraction rules defined by the algorithm developers after a careful and profound investigation on a set of clinical data [6]. It is difficult to achieve perfection, because some features are hidden and hard to discern. This factor undermined the performance of traditional automated cerebral infarct delineation algorithms. In contrast to traditional automated methods, artificial intelligence methods based on deep neural networks can learn image features from the training data [10]. They can potentially facilitate the cerebral infarct modeling and alleviate the limiting factor of traditional non-deep learning methods [11]. In particular, the convolutional neural networks (CNN), a class of artificial neural network, use convolution kernels to extract specific image features. They have been successfully applied in different image classification problems [12].

Motivated by the need for improving the prognosis and treatment of acute ischemic strokes, this research developed a CNN-based automated method to facilitate a facile and quick cerebral infarct detection on brain CT images. Image preprocessing, statistical analysis-based detection, and data augmentation were used to enhance the detection performance of the developed CNN.

## 2. Materials and Methods

### 2.1. Image Data

#### 2.1.1. Subjects

This research utilized radiological cerebral imaging data of 59 subjects with acute ischemic stroke recruited with informed consent at Landseed International Hospital, Taoyuan, Taiwan. The recruited subjects included 35 males and 24 females. They aged between 32 and 74 and averaged 60 years of age. The protocol of this research had been reviewed and approved by the Institutional Review Board (IRB) of Landseed International Hospital. The collected radiological imaging data included CT images and MRI of each subject’s head scan. The average time from an individual subject’s CT scan to MRI scan was 108.81 h. The purpose of collecting the MRI data was mainly to provide more precise cerebral infarct location information to facilitate the training and testing of the CNN. The cerebral infarct regions of the collected MRI’s were delineated by an experienced neurologist (Chen YW) [8,13]. Among the 59 recruited subjects, 38 with cerebral infarction so minor that their CT images contained no trace of infarct were picked up to form a control group. The remaining 21 recruited subjects formed a patient group, from which 16 were randomly selected for the training set and the other five were the test set for the CNN modeling.

#### 2.1.2. Image Acquisition Protocol

A 24-row GE BrightSpeed S CT scanner manufactured by GE Medical Systems (Chicago, IL, USA) was used to acquire all the CT images. The CT scan was performed at 120-kV tube voltage. A standard convolution kernel was used for the reconstruction of CT images. The number of slices for each subject was 28. Each slice contained 512 × 512 voxels with a voxel size of 0.49 × 0.49 × 5 mm^3^. 

A Signa HDxt 1.5T Optima edition (GE Healthcare, Waukesha, WI, USA) was used to acquire all the MRIs, including the diffusion-weighted magnetic resonance images (DWIs) (repetition time (TR) = 6000 ms, echo time (TE) = 82.8 ms, flip angle = 90°, field of view (FOV) = 230 × 230 mm^2^, matrix = 128 × 128, in-plane resolution = 1.79 × 1.79 mm^2^, slice number = 24, slice thickness = 5 mm, slice gap = 1 mm), the T1w sequence (TR = 2400 ms, TE = 24 ms, echo train length (ETL) = 6, FOV = 230 mm, number of excitations (NEX) = 2, matrix size = 288 × 192, slice thickness = 5 mm, and slice gap = 1 mm), and the apparent diffusion coefficient (ADC) map (b = 1000 s/mm^2^).

### 2.2. Image Preprocessing

The preprocessing of the CT images was implemented on MATLAB (MathWorks, Inc., Natick, MA, USA) with applications of the Statistical Parametric Mapping program, SPM8 (Functional Imaging Laboratory, Institute of Neurology, University College London, London, UK). The CT images of the control group and those of the patient group had to go through different preprocessing steps, as described below in Section 2.2.1 and Section 2.2.2, respectively. Moreover, image processing of the patients’ MRI is described in Section 2.2.3.

#### 2.2.1. Preprocessing of the Control CT Images

In this phase, the control CT images were first processed to establish our own CT template in the MNI (Montreal Neurological Institute) space that was supposed to be more suitable for the local subjects than other MNI templates were, considering the difference in the CT protocols and the brain shapes between the local subjects and the subjects involved in the creation of MNI templates. Furthermore, an average CT map and a standard deviation map that represented the descriptive statistics of the control CT images were constructed. Phase One comprised Step C1–C7, as described below.

*Step C1.* DICOM to NIfTI conversion

The original DICOM file format of the control CT images was converted into the three-dimensional NIfTI-1 (Neuroimaging Informatics Technology Initiative) file format. Since SPM8 uses NIfTI-1 as the file format of image data, this conversion facilitated the subsequent image preprocessing with SPM8.

*Step C2.* Resetting the CT image orientation

The origin of the CT images after Step C1 were shifted to roughly align to their anterior commissure of the individual brain space. The purpose of this step was to raise the performance of normalizing the CT images to the standard brain space with SPM8 in next step.

*Step C3.* Spatial normalization to the MNI space

The CT images after Step C2 were spatially aligned to the MNI152 template of MNI space using the normalization tool of SPM8. The dimension of each control CT changed from the original 512 × 512 × 28 voxels to 181 × 217 × 181 voxels after this step.

*Step C4.* Pixel intensity transformation

The pixel intensities of the CT images after Step C3 were piecewise transformed using invertible formulas as proposed by Rorden et al. [14]. The pixel intensities were in Hounsfield unit (HU). HU from −1000 to −100 was transformed to 0–900 with the formula HU + 1000. HU from −99 to 100 was transformed to 911–3100. HU from 101 to 1000 was transformed to 3101−4000 with the formula HU + 3000.

*Step C5.* Establishing our own CT template in MNI space

All the control CT images after Step C4 were voxel-wise averaged across subjects to form our own CT template in MNI space.

*Step C6.* Spatial normalization to our own CT template 

The CT images after Step C2 were spatially normalized to our own CT template established in Step C5. Notice that this step was similar to Step C3, except that the target template was our own CT template instead of the MNI152 template.

*Step C7.* Pixel intensity transformation

This step was the same as Step C4, except that the input of this step was the control CT images after Step C6.

*Step C8.* Elimination of the skull and CSF

The mean and standard deviation of the voxel intensity in the whole brain after Step C7 were calculated to reveal the probability distribution of voxel intensity. Ventricles and brain contour were segmented by thresholding with (mean − 2 × standard deviation) and (mean + 2 × standard deviation), respectively. After applying ventricle and brain masks, the resulted CT image contained only the brain parenchyma, without the CSF or the skull.

*Step C9.* Spatial smoothing of the normalized CT image

The CT images after Step C8 were spatially smoothed with a spatially stationary Gaussian filter. To maximize the detection performance, we adopted the evaluation result done by Gillebert et al. [4] and chose the size of the Gaussian smooth kernel to be 5-mm FWHM (Full-Width at Half-Maximum). 

*Step C10.* Establishing the average CT map and the standard deviation CT map

The average CT map and the standard deviation CT map constituted a voxel-specific statistic description of all the control CT images. In the average CT map, each voxel was assigned the average of the preprocessed values at all the corresponding voxels of the control CT images. Similarly, in the standard deviation CT map, each voxel was assigned the standard deviation of the preprocessed values at all the corresponding voxels of the control CT images.

#### 2.2.2. Preprocessing of the Patient CT Images

The purpose of this preprocessing was to construct a t-score map for each patient. It comprised Steps P1–P7, as described below. Some of these steps were the same as some of the preprocessing steps of the control CT images described in Section 2.2.1.

*Step P1.* DICOM to NIfTI conversion

This step was the same as Step C1, except that the input of this step was the patient CT images.

*Step P2.* Resetting the CT image orientation

This step was the same as Step C2, except that the input of this step was the patient CT images after Step P1.

*Step P3.* Normalizing the patient’s CT images to our own CT template

This step was similar to Step C6, except that the input of this step was the patient CT image after Step P2. The dimension of each patient CT changed from the original 512 × 512 × 28 voxels to 181 × 217 × 181 voxels after this step, resulting in a voxel-to-voxel correspondence between the patient’s CT image and our own CT template. This enabled a cerebral infarct detection based on a statistical analysis of patient’s CT image with reference to the control CT images.

*Step P4*. Pixel intensity transformation 

This step was the same as Step C4, except that the input of this step was the patient CT images after Step P3.

*Step P5*. Elimination of the skull and CSF

This step was the same as Step C8, except that the input of this step was the patient CT images after Step P4.

*Step P6.* Spatial smoothing of the normalized CT image

This step was the same as Step C9, except that the input of this step was the patient CT image after Step P5.

*Step P7*. Constructing the t-score map

After the individual patient’s CT images had gone through the above steps, a t-score would be evaluated for each voxel. The collection of the t-scores of the whole-brain voxels is called the t-score map of the individual patient. A t-score reveals the relative intensity of a patient voxel with respect to the probability distribution of the corresponding control voxels across all the control subjects. Referring to the method presented by Gillebert et al. [4] and Crawford et al. [15], the t-score of a patient voxel was defined as:

(1) $$t=\frac{p-{\bar{X}}_{C}}{\sqrt{\left(n+1\right)/n}\times s_{C}}$$

where *p* was the intensity of the voxel of the individual patient, n was the sample size of the control group, and ${\bar{X}}_{C}$ and $s_{C}$ were the average and the standard deviation of the intensity, respectively, of the corresponding voxels across all the control subjects. The values of ${\bar{X}}_{C}$ and $s_{C}$ were taken from the control group’s average CT map and standard deviation CT map, respectively, which had been established in Step C10. In Equation (1), the denominator contained a correction factor $\sqrt{\left(n+1\right)/n}$ to account for the uncertainty of the control mean and standard deviation.

#### 2.2.3. Infarct Segmentation and Spatial Normalization on MRI

The training of the proposed CNN required training data of both non-infarcted and infarcted CT images. Semi-automated infarct segmentation on the collected MRIs was conducted by the experienced neurologist (Chen YW) to provide precise infarct location information. Furthermore, the segmented infarcted regions must be normalized to a standard template (common to that for standard CT template). The following steps describe the image processing on the MRIs.

*Step M1.* DICOM to NIfTI conversion

The original DICOM file format of the MRIs was converted into the three-dimensional NIfTI-1 file format to facilitate the subsequent image preprocessing with SPM8.

*Step M2.* Infarct segmentation on the DWIs

The experienced neurologist (Chen YW) conducted semi-automated infarct segmentation on the DWIs. The ADCs were referred to in this step to eliminate artifacts.

*Step M3.* Resetting the DWI orientation

The origin of the DWIs after Step M1 were shifted to roughly align to their anterior commissure of the individual brain space. The purpose of this step was to raise the performance of normalizing the DWIs to the standard brain space with SPM8 in the next step.

*Step M4.* Spatial normalization to the MNI space

The DWIs after Step M3 were spatially aligned to the MNI152 DWI template of MNI space using the normalization tool of SPM8.

*Step M5.* Establishing our own DWI template in MNI space

All the DWIs after Step M4 were voxel-wise averaged across subjects to form our own DWI template in MNI space.

*Step M6.* Spatial normalization to our own DWI template

The DWIs after Step M3 were spatially normalized to our own DWI template established in Step M5. Notice that this step was similar to Step M4, except that the target template was our own DWI template instead of the MNI152 template.

### 2.3. Infarct Detection

#### 2.3.1. CNN Structure

**Figure 1:**
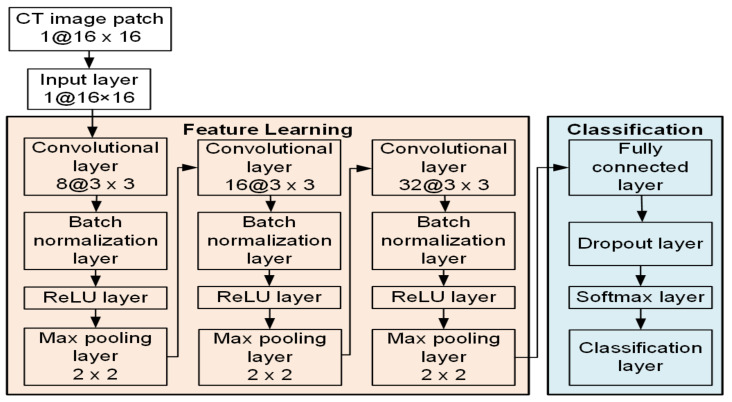
Schematic illustration of the proposed CNN architecture for the patch-wise classification task.

The CNN architecture we proposed in this research consisted of 17 layers, including an input layer, three convolutional layers, three batch normalization layers, three rectified linear unit (ReLU) layers, three max pooling layers, a fully connected layer, a dropout layer, a Softmax layer, and a classification layer (output layer). Figure 1 shows the architecture of our proposed CNN structure.

#### 2.3.2. Training and Testing of CNN

The convolutional neural network was trained with the training data taken from the t-score maps, which were the output of the image preprocessing. The input to the CNN was in the form of a matrix of 16 × 16 t-scores pertaining to 16 × 16 contiguous pixels in a square area on a CT slice. The training data consisted of t-score matrices of both infarcted and non-infarcted patches. The infarcted patches were taken on patient CT areas whose corresponding MRI areas contained some infarcted pixels, as was known by referring to the corresponding MRI slices that had been segmented and spatially normalized as described in Section 2.2.3. The non-infarcted patches were taken on the control CT areas at locations roughly matching the locations of the infarcted patches.

**Figure 2:**
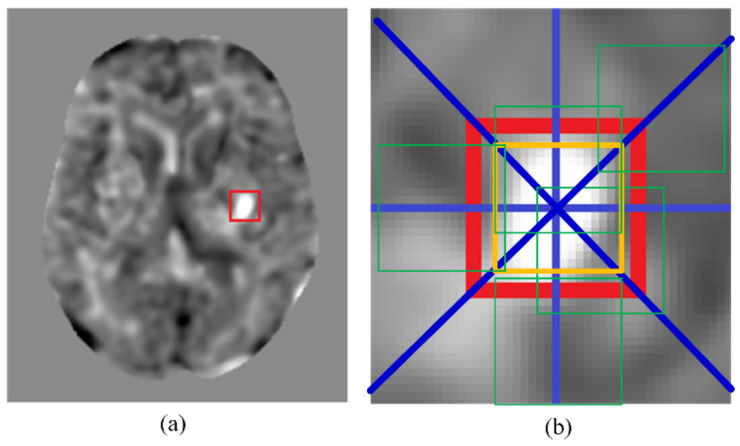
An example of data augmentation. (**a**) A t-score map with an infarcted region in the red box. (**b**) The golden box represents the initial patch location. The green boxes are examples of additional patches due to the data augmentation. The blue lines show the eight shifting directions to create the additional patches.

A large amount of data is needed for the training of a convolutional neural network. Training with insufficient data may lead to overfitting and compromise the detection accuracy of the network. However, the collected clinical data, especially infarcted data, usually will not suffice the required amount for CNN training. To solve this problem, we conducted data augmentation to increase the size of the training dataset. Figure 2 illustrates how data augmentation of an infarcted patch was conducted. Shown in Figure 2a is a t-score map that contains an infarcted region in the red box. In Figure 2b, the golden box demarcates the original infarcted patch. The eight blue lines emanating from the center of the original patch represent the eight directs, along which the center of the original patch was shifted to define additional patches. Data augmentation of non-infarcted patches was also conducted in the same way. An additional patch would not be adopted if the shifting altered it from an infarcted patch to a non-infarcted patch or vice versa. An additional patch must also remain parenchyma-bounded to be adopted. An additional patch containing pixels in the skull or in the ventricle would not be adopted. After data augmentation, there were 2656 infarcted patches available. With the addition of 2826 randomly picked non-infarcted patches, the training dataset contained 5482 patches. Among these patches, a randomly selected 80% (4386 patches) were used for the CNN training and the remaining 20% (1096 patches) were used for validation. By resampling the training set and the validation set, cross validation was repeatedly conducted for five times. Note that about the same amounts of infarcted and non-infarcted data were used for the network training, despite the infarcted regions possibly only taking up a small portion of the entire brain. This was to avoid imbalanced training, which could result in a network model that favored the classification accuracy of the majority class and might lead to misclassification of the minority class.

The stochastic gradient descent with momentum (SGDM) optimizer was used for training CNN, and the loss function in the classification output layer was a cross entropy function. The initial and final learning rates of the CNN model were set to 10^−5^ and 10^−7^, respectively. The maximum epoch was 1000, the maximum iterations was 8000, the momentum coefficient was 0.9, and the size of the mini-batch to use for each training epoch was 500. In addition, we used the dropout regularization in the fully connected layer in order to avoid overfitting. The dropout ratio was set to 0.5 and the weight decay was set to 0.005.

#### 2.3.3. Clinical Application

When applying the trained CNN for clinical cerebral infarct detection, each cerebral CT slice of about 181 × 217 pixels will be divided into about 153 nonoverlapped patches of 16 × 16 pixels. After deleting patches containing pixels outside of the parenchyma or in the ventricle, about 13,000 patches will be obtained from the 181 slices of a patient. Each of these patches will be independently detected with the trained CNN to check for the existence of cerebral infarction. Cerebral hemorrhage and infarct detection on a patient suspected of a stroke is crucial for accurate treatment decision making. Detection of cerebral hemorrhage from CT images is relatively easier. However, detection of cerebral infarct is not as easy. The cerebral infarct detection with the trained CNN could attain a high accuracy rate and would be faster than the traditional automated infarct detection methods, making it suitable for emergent detection of cerebral infarct when CT is available.

## 3. Results

**Figure 3:**
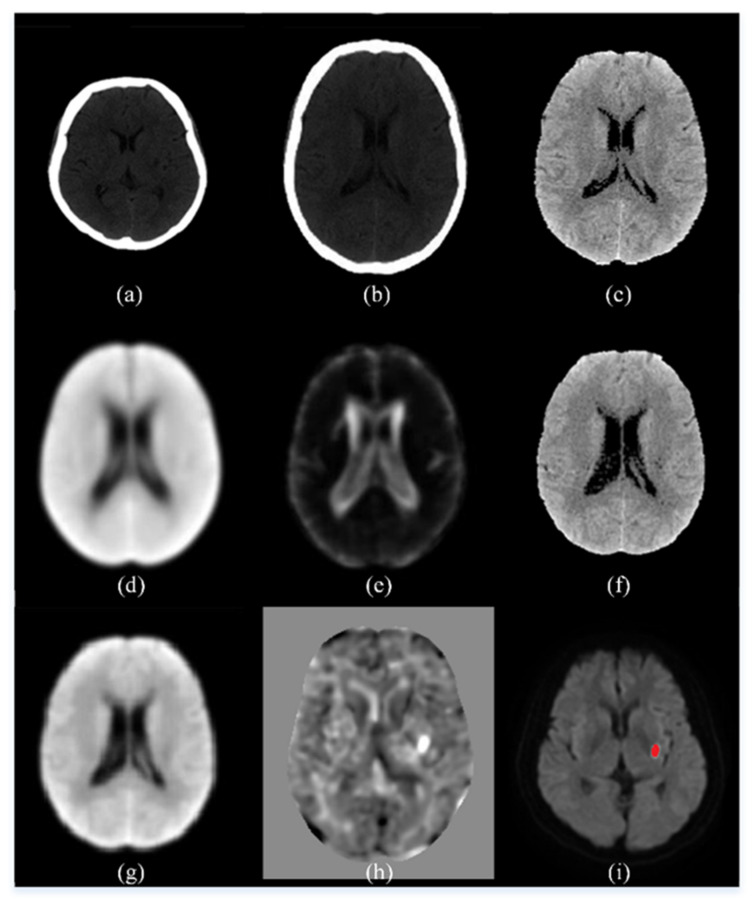
An illustration of the intermediate images in the image preprocessing steps. (**a**) An original control CT image; (**b**) the result of the spatial normalization; (**c**) the result of intensity transformation; (**d**) the average CT map of all control CT; (**e**) the standard deviation CT map of all control CT; (**f**) a patient CT image after normalization to our own CT template; (**g**) the result of spatial smoothing by the kernel with a 5-mm FWHM; (**h**) the t-score map; (**i**) the ischemic infarct area drawn by the experienced neurologist.

Shown in Figure 3 are some intermediate images produced during the preprocessing steps. The images in the top row are, from left to right, an original control CT image, the result of the spatial normalization (Step C3), and the result of intensity transformation (Step C4). The images in the central row are, from left to right, the average CT map of all control CT (Step C10), the standard deviation CT map of all control CT (Step C10), and a patient CT image after normalization to our own CT template (Step P3). The images in the bottom row are, from left to right, the result of spatial smoothing by the kernel with a 5-mm FWHM (Step P6), the t-score map (Step P7), and the ischemic infarct area drawn by the clinician.

The result of training and testing the proposed CNN is shown in the confusion matrices in Table 1. The performance of the CNN is evaluated in terms of the accuracy rate, defined as:

(2) $$\mathrm{Accuracy}\;\mathrm{rate}=\frac{TP+TN}{TP+TN+FP+FN}$$

where TP, TN, FP, and FN stand for true positive, true negative, false positive, and false negative, respectively. Table 1 indicates that the proposed CNN attained a good accuracy rate of 94.4% in the training set. The accuracy rate in the test set was 93.9%.

**Figure 4:**
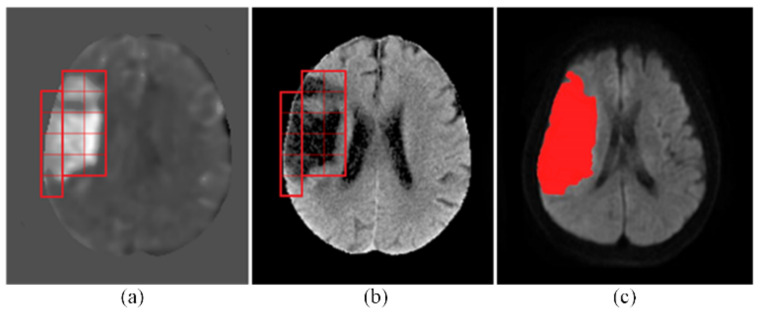
An exemplary result of infarct detection by the proposed CNN. (**a**) The detected infarcted patches were marked with red squares on the t-score map. (**b**) They were also marked on the intensity-transformed brain parenchyma image, i.e., the CT image after Step P5. (**c**) The red area on the MRI represents the detected infarcted region delineated by the experienced neurologist.

The output of cerebral infarct detection with the proposed CNN is exemplified in Figure 4. The detected infarcted patches on a CT slice are marked by red squares on the t-score map of the CT slice in Figure 4a, whereas, in Figure 4b, they are marked by red squares on the intensity-transformed brain parenchyma image, i.e., the CT image after Step P5. Shown in Figure 4c is the corresponding MRI slice on which the red area represents the detected infarct delineated by the experienced neurologist mentioned above (Chen YW) [13] with a semi-automated method.

**Figure 5:**
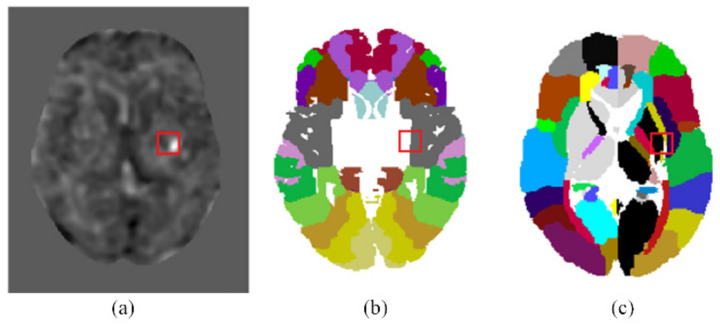
Mapping the detected infarcted patches onto the Brodmann areas and EVE atlas template. (**a**) The location of an infarcted patch detected by the proposed CNN on the t-score map. (**b**) The location of the infarcted patch is mapped onto the Brodmann areas. (**c**) The location of the infarcted patch is mapped onto the EVE atlas template.

Using the coordinates of the detected infarcted patches on the t-score map, we can identify the corresponding brain areas on Brodmann areas or Eve Atlas. For example, Figure 5a shows a detected infarcted patch on the t-score map. This detected infarct corresponded to an acute infarction in the right middle cerebral artery (MCA) territory delineated by the experienced neurologist. Figure 5b shows that this detected infarcted patch covered a portion of area 48 (Retrosubicular area) of the Brodmann areas, which is a small part of the medial surface of the temporal lobe. Figure 5c shows that it involved areas EA25 (INSULAR), EA47 (External_capsule_left), EA60 (PUTAMEN_left), and EA62 (GLOBUS_PALLIDUS_left) on the Eve Atlas. Note that the patient CT images were spatially normalized to MNI space in Step P3 and the Brodmann areas and EVE Atlas had also been normalized to the MNI space. A detected infarcted patch had the same coordinates on the patient’s CT image, the Brodmann areas, and the EVE Atlas. Hence, mapping a detected infarcted patch to the Brodmann areas or EVE Atlas was conducted simply by using the same coordinates and required no image processing.

The proposed method was implemented with MATLAB programming. The MATLAB program takes about 5 min per patient from reading the image files until finishing the infarct detection. The program ran under Microsoft Windows 10 in a desktop computer with an Intel Core i7 CPU and an 8-gigabyte RAM, without a GPU (graphics processing unit).

## 4. Discussion

The performance of the automated cerebral infarct delineation on MRI has been proved to render accurate results due to the high contrast of MRI [8]. However, MRI is normally not readily available immediately following the stroke onset. On the contrary, CT is a more convenient modality that can be utilized short after the stroke onset. The result of this research shows that by incorporating the power of deep learning with CNN, the accuracy of the automated cerebral infarct detection on CT could attain an acceptable level (93.9% in the test set). Hence, the automated cerebral infarct detection on CT can potentially be good enough for a frontline detection of cerebral infarction, despite the lower contrast of CT images. 

In this research, the patch size was selected after considering the accuracy rate, detection time, and even network training time. Training time was quite tolerable in this research, because our simple network did not require a long training time. The most important factor to consider would be the accuracy rate. Among the 32 × 32, 16 × 16, and 8 × 8 patch sizes, the 16 × 16 patch size led to 93.9% accuracy rate and the 8 × 8 patch size is on par with it, whereas the 32 × 32 patch size only led to an accuracy rate over 80%. The detection time affects the end user’s working efficiency and convenience and is, hence, also important. We first supposed that it would be the fastest with the 8 × 8 patch size. However, in fact, the fastest was with the 16 × 16 patch size, which was about 50% faster than with the 8 × 8 patch size. The reason was because the patch number extracted from a patient’s CT images with the 8 × 8 patch size was four times that with the 16 × 16 patch size. Thus, the 16 × 16 patch size was a better selection than the other two sizes. 

With the proposed method, a large infarct shown as the red area in Figure 4c was faithfully detected as a mosaic of 15 contiguous tesserae (patches) shown in Figure 4a,b. The shapes of the true infarct in the individual tesserae were versatile, as can be seen in Figure 4a. In the scope of some patches, the infarct took the whole patch area, whereas in some others patches, the infarct took a small area. The infarct shapes in different patches were different. This detection result has demonstrated the capability of the proposed CNN in detecting infarcts of various morphologies. This result has also confirmed that the 16 × 16 patch size was truly an adequate selection for the proposed CNN. Moreover, this result has also corroborated the sufficiency of the training data by data augmentation fulfilled with only the shifting operation, without using other operations, such as rotation, flipping, cropping, padding, or intensity transforms.

As stated in Section 2.1.1, infarcted images of 16 recruited subjects and non-infarcted images of 38 recruited subjects were used for the training of the proposed CNN. One may question whether the number of subjects were too small and, hence, whether the proposed network could be insufficiently trained. In fact, the training and the infarct detection of the proposed CNN was patch-wise instead of slice-wise or subject-wise. After data augmentation on the original patches, there were totally 4176 patches for the training of the proposed CNN. Thus, the amount of the training dataset was sufficiently large, as has been evidenced by the high accuracy rate of our trained CNN.

**Figure 6:**
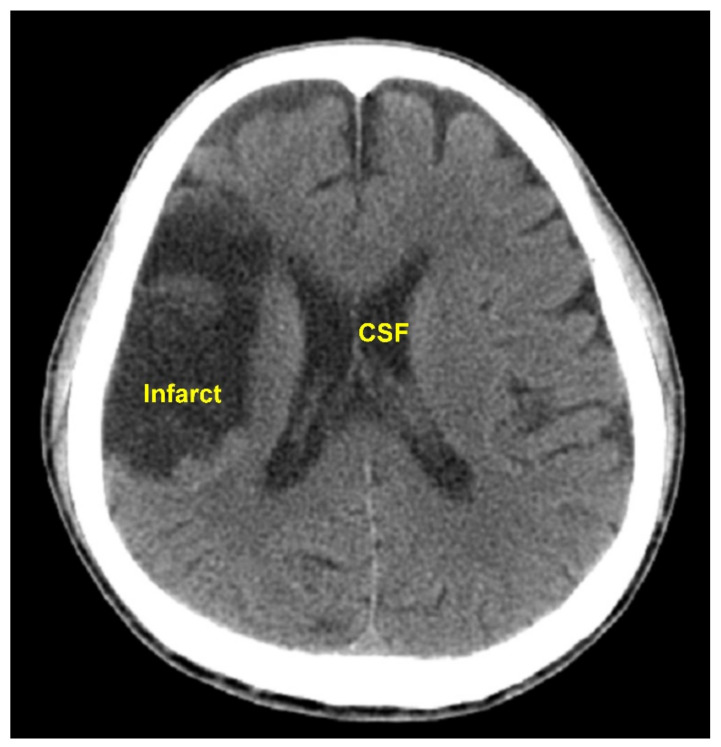
This CT image shows similar intensities of the CST and the infarct region. The CST could be falsely detected as infarcted if not eliminated before the infarct detection.

The pixel intensity transformation carried out in Step C4, Step C7, and Step P4 changed the HU of every pixel. It was an important image preprocessing function. The performance of our method would not have been satisfactory without this effort. The approximate HUs of air and bone are −1000 and 1000, respectively. The pixels in the intracranial tissues have HUs near 0. For example, the HU of CSF = 0, white matter = 25, gray matter = 35, and blood = 60 [14]. In Steps C4, C7, and P4, the distance between two consecutive HUs in the range −99 to 100 was enlarged by 11 times. Thus, the contrast of different tissues in the brain were enhanced. On the other hand, the HUs from −1000 to −100 and from 101 to 1000 only received a constant raise without a contrast enhancement. Another effort of the image preprocessing that contributed to the high accuracy rate was the elimination of the CSF, carried out in Step C8 and P5. CSF could have intensities close to those of the cerebral infarcts and might lead to false positive results. For instance, in the CT image shown in Figure 6, the intensity in the CST was close to that in the infarct region. The CST would be falsely detected as infarcted if it was not eliminated.

There is a possible change with time in the boundary line of an infarcted region in the brain past stroke onset, so the radiological images taken at different times after stroke may have different detectable cerebral infarct boundaries. For example, the manifestation of an infarcted cerebral region on the DWI becomes brighter in the first several days after the onset, and it gradually turns darker in the following several days. In this retrospective research, there was a delay of 3.8 ± 1.5 (mean ± standard deviation) days between a recruited subject took CT and MRI scans. In this research, in order to use supervised learning to train the proposed CNN for detecting cerebral infarcts from CT images, the results of the semi-automated MRI infarct delineation by the experience neurologist mentioned above were taken as the correct output for the network training. Due to the time delay of taking an MRI scan after taking a CT scan, there was a discrepancy between the detected cerebral infarct boundary on the MRI and the real cerebral infarct boundary at the time of CT scan. This discrepancy became a source of error for network training and a limit to the accuracy rate of the proposed CNN. 

**Figure 7:**
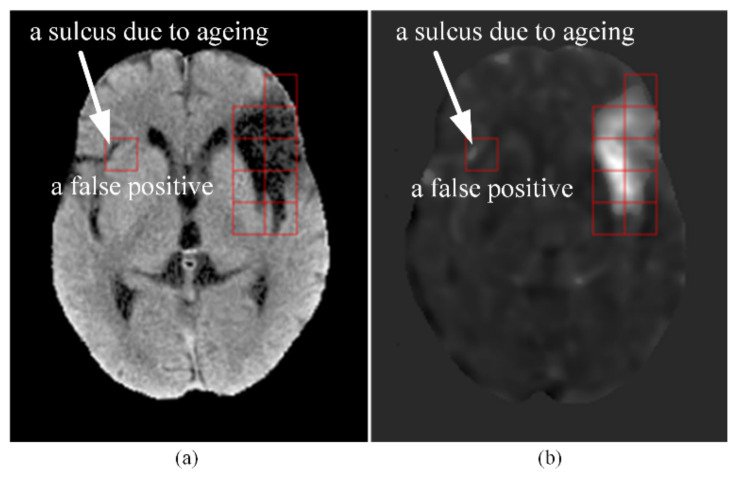
An example of a false positive caused by age mismatch between the control group and the patient group. (**a**) An elderly patient’s CT slice after Step P5. The dark area inside the red square on the right hemisphere was non-infarcted and was a sulcus due to ageing. (**b**) The t-score map of the CT slice after Step P7. Notice the high t-scores in the sulcus encompassed by the red square on the right hemisphere, which led to a false positive detection of this patch.

Brain atrophy can happen with ageing in forms of general or focal shrinkage of brain structure [16]. However, it is usually not easy to attain age matching of the control group and the patient group. As described in Section 2.2 (Step P7), the t-score of each pixel of the patients was obtained by using the probability distribution of all the control CTs as the reference. Hence, the accuracy of the cerebral infarct detection might be affected by the ageing effect. In other words, the false detection (false positive or false negative) might be due to the unmatched ages between the patient group and the control group. Shown in Figure 7 is an example of the result of cerebral infarct detection with our method on the CT of an elderly patient from the test set. In Figure 7a, the red square on the right hemisphere encompassed a darker area that was actually the image of a sulcus formed due to ageing and filled with fluid. As shown in Figure 7b, this sulcus location of the patient got high t-scores when its intensities were compared with the average intensities at the corresponding location of the control group, where there was no sulcus. This caused the patch to be falsely detected as an infarcted patch. To alleviate this problem, it is important to implement an age match between the control group and the patient group. Provided enough subjects can be recruited, a better strategy is to form several control groups of different age ranges and establish different average CT maps and standard deviation CT maps (in Step C10) for patients of different age groups.

Physicians have been applying grading systems as a basis for decision making after stroke onset. ASPECTS is a widely used means for evaluating early ischemic changes in acute strokes [17]. The scoring is based on non-contrast CT scans. To compute the ASPECTS, 1 point is subtracted from 10 for any evidence of early ischemic change in any of the 10 defined regions. In fact, ASPECTS evaluation does not cover all the cerebral areas and sometimes the physicians may want to know the cerebral infarct location in terms of a more precise brain map. Figure 5 exemplifies mapping the detected cerebral infarct patches to two popular brain maps with finer brain parcellation. The Brodmann’s map, as shown in Figure 5b, is the most popular cortical map. In this map, the human cerebral cortex is divided into 52 areas on the basis of the observations of the cytoarchitecture. Each area is assigned a number [18]. The JHU-MNI-ss atlas, also called the Eve atlas, as shown in Figure 5c, from Johns Hopkins University, emphasizes the parcellation of the white matter, while also containing the grey matter. It is a single-subject female brain with an isotropic resolution of 1 mm^3^ in the standard MNI coordinates [19].

It was found that the infarct volume has a correlation with the National Institutes of Health Stroke Scale (NIHSS) [20]. Hence, including NIHSS in the training data could possibly enhance the network training effectiveness and raise the detection accuracy. NIHSS is used to evaluate the level of neurological deficits due to acute cerebral infarction. It is scored by rating the patient’s ability in answering questions and performing activities. A trained observer requires less than 10 min to complete the rating of the 15-item neurologic examination of NIHSS. Considering the short rating time and the possibly higher detection accuracy, it seems worthwhile to conduct NIHSS rating and incorporate NIHSS in the network training.

A literature review has found few papers similar to this work that applied deep learning with CNN for cerebral infarct detection on brain CT. Tuladhar et al. [21] developed a CNN-based infarct segmentation method and attained a Dice similarity coefficient (DSC) of 0.45%. The generalization of their model was strengthened by using independent multi-center datasets for training, test and validation, as well as using ground truth segmentations by multiple expert observers. Sales Barros et al. [22] utilized a three-CNN approach for cerebral infarct segmentation and attained a 0.57 average DSC of voxel-wise accuracy and a 0.88 intraclass correlation coefficient (ICC) of infarct volume. The three CNNs were developed for the delineation of subtle, intermediate, and severe infarcts, respectively. Gautam et al. [23] used CNN to classify brain CT into hemorrhagic stroke, ischemic stroke, or normal and attained a 92.22% classification accuracy.

The most valuable application of the automated CT cerebral infarct detection will be for the prompt detection of cerebral infarction using the CT taken in the emergency room. As the phrase “time is brain” emphasizes, the brain nerves quickly lose function when a stroke occurs. Incorporating the proposed CNN for the accurate real-time CT-based cerebral infarct detection can help save brain function in time and enhance the efficiency of stroke care.

## 5. Conclusions

In this research, we developed a fully automated method for cerebral infarct detection from CT images. The convolutional neural network structure was adopted to deliver artificial intelligence that is capable of extracting image features from the CT data and classifying image patches of CT images into infarcted or non-infarcted ones. Data augmentation supplemented the limited amount of clinical data to ensure sufficient training of the convolutional neural network, which prevented overfitting and enhanced the detection accuracy. Image preprocessing on the patient data and the control data emphasized the critical information pertaining to successful cerebral infarct detection from CT images. The CNN attained about 93.9% accuracy in cerebral infarct detection from CT images in the test set. The proposed CNN-based detection method could provide an assistance in the first-step diagnosis of cerebral infarction with CT scan, which is low-cost and readily available.

## Figures and Tables

**Table 1:** Evaluation of the proposed method.

	Training Set		Test Set	
	Positive	Negative	Positive	Negative
Infarct	TP = 46.81%	FN = 0.87%	TP = 46.26%	FN = 0.73%
Non-infarct	FP = 4.72%	TN = 47.61%	FP = 5.38%	TN = 47.63%
Accuracy rate	94.4%		93.9%	
Sensitivity	98.2%		98.4%	
Specificity	91.0%		89.8%	

Note: TP = True Positive, FP = False Positive, TN = True Negative, FP = False Negative.

## Data Availability

Not applicable.
